# Mononuclear Phagocytes Are Dispensable for Cardiac Remodeling in Established Pressure-Overload Heart Failure

**DOI:** 10.1371/journal.pone.0170781

**Published:** 2017-01-26

**Authors:** Bindiya Patel, Mohamed Ameen Ismahil, Tariq Hamid, Shyam S. Bansal, Sumanth D. Prabhu

**Affiliations:** 1 Division of Cardiovascular Disease, University of Alabama at Birmingham, Birmingham, AL, United States of America; 2 Comprehensive Cardiovascular Center, University of Alabama at Birmingham, Birmingham, AL, United States of America; 3 Medical Service, Birmingham VA Medical Center, Birmingham, AL, United States of America; Albert Einstein College of Medicine, UNITED STATES

## Abstract

**Background:**

Although cardiac and splenic mononuclear phagocytes (MPs), *i*.*e*., monocytes, macrophages and dendritic cells (DCs), are key contributors to cardiac remodeling after myocardial infarction, their role in pressure-overload remodeling is unclear. We tested the hypothesis that these immune cells are required for the progression of remodeling in pressure-overload heart failure (HF), and that MP depletion would ameliorate remodeling.

**Methods and Results:**

C57BL/6 mice were subjected to transverse aortic constriction (TAC) or sham operation, and assessed for alterations in MPs. As compared with sham, TAC mice exhibited expansion of circulating LyC6^hi^ monocytes and pro-inflammatory CD206^−^ cardiac macrophages early (1 w) after pressure-overload, prior to significant hypertrophy and systolic dysfunction, with subsequent resolution during chronic HF. In contrast, classical DCs were expanded in the heart in a biphasic manner, with peaks both early, analogous to macrophages, and late (8 w), during established HF. There was no significant expansion of circulating DCs, or Ly6C^+^ monocytes and DCs in the spleen. Periodic systemic MP depletion from 2 to 16 w after TAC in macrophage Fas-induced apoptosis (MaFIA) transgenic mice did not alter cardiac remodeling progression, nor did splenectomy in mice with established HF after TAC. Lastly, adoptive transfer of splenocytes from TAC HF mice into naïve recipients did not induce immediate or long-term cardiac dysfunction in recipient mice.

**Conclusions:**

Mononuclear phagocytes populations expand in a phasic manner in the heart during pressure-overload. However, they are dispensable for the progression of remodeling and failure once significant hypertrophy is evident and blood monocytosis has normalized.

## Introduction

Pathological left ventricular (LV) remodeling is a hallmark of chronic pressure-overload. With the imposition of augmented pressure load, the LV initially remodels with concentric hypertrophy and preserved systolic function, but over time transitions to a phenotype of dilated cardiomyopathy and systolic heart failure (HF). The development of pressure-overload HF is accompanied by structural and metabolic changes within the myocardium, including cardiomyocyte hypertrophy, interstitial fibrosis, inflammation, and a switch toward a fetal-like metabolic profile [1–4]. While HF secondary to pressure-overload is fundamentally a direct result of increased hemodynamic load and wall stress, the underlying tissue-level mechanisms contributing to disease progression are not clearly understood.

Previous studies in mice have demonstrated that during pressure-overload, inflammatory cytokines such as tumor necrosis factor-α (TNF), interleukin (IL)-4, and IL-6 are important disease drivers that promote cardiac fibrosis, hypertrophy, and dysfunction [5–7]. Moreover, patients with pressure-overload exhibit augmented myocardial expression of TNF, IL-1β, and IL-6 [8]. Mononuclear phagocytes (MPs)—innate immune cells comprised of monocytes, macrophages, and dendritic cells (DCs)—orchestrate the inflammatory response by releasing cytokines and proteases, effecting phagocytosis and efferocytosis, and activating myofibroblasts and immune cells [9, 10], suggesting that MPs may contribute to cardiac pathology in pressure-overload HF. However, while macrophage abundance has been shown to increase in the pressure-overloaded heart [5, 11, 12], the recruitment characteristics and functional importance of these cells during chronic pressure-overload HF has not been well studied.

In non-reperfused and reperfused murine myocardial infarction models, we and others have identified distinct innate immune cell populations that modulate both early and late cardiac remodeling after infarction [13–16]. Moreover, in chronic ischemic HF, we have identified a pathological cardiosplenic axis, whereby innate immune cells traffic from the spleen to the heart to induce myocardial tissue injury [16]. However, whether and how splenic immune cells influence cardiac remodeling in non-ischemic forms of HF, such as engendered by mechanical load, is unknown. Accordingly, we characterized alterations in MPs in the heart and spleen during pressure-overload, and tested the hypothesis that these immune cells play an essential role in the progression of cardiac remodeling in chronic pressure-overload HF.

## Methods

### Mice

Wild-type (WT) male CD45.1 (#002014) and CD45.2 (#000664) C57BL/6 mice, and macrophage Fas-induced apoptosis (MaFIA) transgenic mice (#005070) were purchased from Jackson Laboratories. All mice were maintained at the University of Alabama at Birmingham (UAB); the UAB Institutional Animal Care and Use Committee approved all the mouse studies described. All surgeries were performed in 9–12 week-old mice. A total of 125 mice were used for these studies.

### Transverse aortic constriction (TAC)

TAC was used to induce pressure-overload as previously described [17]. Briefly, under inhalation anesthesia with 1.5% isoflurane in 100% O_2_, mice were intubated and mechanically ventilated with a tidal volume of 200 μL and rate of 150 breaths/minute (Harvard Apparatus MiniVent 845). A limited midline sternotomy was performed from the suprasternal notch to the second rib to expose the aortic arch. A 6.0 silk suture was looped around the aorta between the innominate and left carotid arteries and tied against a 27-gauge needle. The needle was then removed, allowing for a reproducible discrete stenosis of the aortic arch. The chest was closed in layers, the mice were extubated upon evidence of spontaneous breathing, and the animals were allowed to recover. Control animals underwent sham surgery with the identical procedure except that the suture was passed around the aorta but not tied. For studies of selective MP ablation, MaFIA mice were treated with 0.5 mg/kg i.v. AP20187 homodimerizer (Clontech) or vehicle (control) every 10 d starting 2 w after TAC or sham surgery.

### Echocardiography

M-mode, 2D, and Doppler echocardiography were performed under 1.5% inhaled isoflurane anesthesia (with 100% supplemental O_2_) using a VisualSonics Vevo770 High-Resolution System and 30 MHz RMV707B scanhead as previously described [16]. Body temperature was maintained at 37.0°C ± 0.5°C and heart rate at 500 ± 50 bpm on a heated, bench-mounted adjustable rail system. In addition to standard cardiac imaging, the pressure (P) gradient across the constriction was measured using pulse wave Doppler velocity (V) (ΔP = 4V^2^). Only mice with a pressure gradient >50 mm Hg were included in the TAC group. Data analysis was performed using VisualSonics software (VisualSonics).

### Mononuclear cell isolation

Mononuclear cells were isolated from the peripheral blood, heart, and spleen as previously described [16]. Peripheral blood (~200 μL) was collected in EDTA tubes (BD Biosciences). Erythrocytes were lysed with RBC lysis buffer (eBioscience) and the remaining leukocytes were washed with PBS, collected by centrifugation (380*g* for 10 min at 4°C) and resuspended in 400 μL ice-cold flow cytometry staining buffer (eBiosciences). Splenocytes were isolated by flushing the spleen with 3mL of PBS and then filtering the cells through a 100 μm cell strainer (BD Falcon). Cells were centrifuged at 500*g* for 5 min at 4°C and resuspended in staining buffer (eBioscience). For cardiac immune cell isolation, whole hearts were flushed with PBS to remove blood and minced into 2mm pieces using a single-edged blade. The tissue was digested in RPMI media containing collagenase-2 (1mg/mL, Worthington), trypsin (1 mg/mL, Invitrogen), and DNase I (10 ug/mL) at 37°C for 45 min with gentle agitation. Cell suspensions were filtered through a 100 μm cell strainer (BD Biosciences) and incubated with 2 mM EDTA in PBS for 5 min at 37°C. Isolates were then centrifuged at 2000*g* for 20 min on a Ficoll gradient (GE Healthcare), and mononuclear cells were subsequently collected and washed with PBS. Cells were fixed with 1% paraformaldehyde and stored in staining buffer (eBioscience) at 4°C.

### Flow cytometry

Cells were incubated with anti-mouse CD16/32 (BD Biosciences) for 10 min at 4°C to block FcγRIII and FcγRII receptors in 100 μL of staining buffer (eBioscience). Cells were then directly incubated with a cocktail of anti-mouse fluorochrome-conjugated antibodies (eBioscience) in combinations appropriate for the panel of interest: CD45-PerCPCy5.5 (30-F11), CD11b-ef605NC (M1/70), F4/80-ef450 (BM8), MHCII-PE (AF6-120.1), CD11c-PE-Cy7 (N418), CD206-AF647 (MR5D3), Ly6C-PE-Cy7 (HK1.4), NK1.1-PE (PK136), CD90.2-PE (30-H12), Ly6G-PE (RB6-8C5), CD49b-PE (DX5), and CD45R-PE (RA3-6B2). Cells were incubated for 30 min in the dark on ice and subsequently washed with PBS, centrifuged at 500*g* for 5 min at 4°C, and resuspended in PBS. For CD206 staining, cells were permeabilized with 0.5% Tween20 in PBS prior to incubation, washing, centrifugation, and resuspension in PBS. Data were acquired on an LSRII flow cytometer (BD Bioscience) and analyses were performed with FlowJo software, version 10.0.6 (Tree Star).

Blood monocytes were identified from the monocyte-lymphocyte gate as lineage (Lin: CD45R, CD90.2, NK1.1, CD49b) negative, CD11b^+^ cells and were grouped into pro-inflammatory Ly6C^hi^ and patrolling Ly6C^low^ subsets [9, 16, 18]. Cardiac macrophages were identified as CD45^+^CD11b^+^F480^+^MHCII^+^ cells and subclassfied into CD206^–^ pro-inflammatory and CD206^+^ anti-inflammatory macrophages [14, 16, 19]. DCs were identified as Lin (Ly6G, NK1.1, CD90.2, CD49b) negative CD11c^+^ cells. Plasmacytoid DC (pDC) were classified as Lin^–^CD11c^low^B220^+^ [16, 20, 21], and classical DCs (cDCs) as Lin^–^CD11c^+^B220^–^ [16, 21].

### Histological analysis

Formalin-fixed, paraffin-embedded hearts from sham and TAC mice were sectioned at 5 μm thickness, deparaffinized, and rehydrated. Masson’s trichrome staining to evaluate tissue fibrosis was performed as previously described [16, 22, 23] and quantified from 6 high-power fields per section using Metamorph software version 6.3r5 (Molecular Devices).

### Splenectomy and splenocyte adoptive transfer

Male 10 week-old CD45.2 MaFIA mice underwent TAC (n = 10) or sham surgery (n = 10). After 8 w, survival splenectomy was performed on these mice as previously described [16]. Briefly, a laparotomy was performed with a 1 cm incision in the left upper quadrant of anesthetized mice. The splenic hilum was ligated and cauterized, and the spleen removed. Splenocytes were isolated from the spleen under sterile conditions by mechanical disruption and then filtering through a 100 μm cell strainer. Erythrocytes were lysed using RBC lysis buffer (eBiosciences) and the remaining cells were washed with PBS. Splenic mononuclear cells were purified by Ficoll gradient centrifugation. Cells were resuspended in sterile PBS, and pooled from TAC or sham mice. For adoptive transfer, sham or TAC splenocytes (1.1 x 10^7^ cells) were injected via tail vein into naïve CD45.1 recipient 10 week-old male C57BL/6 mice, and the recipients were followed for 8 w. Some recipient mice were injected i.p. with a single dose of either 10 mg/kg AP20187 or vehicle immediately after cell transfer to ablate MPs from donor mice. In addition, cardiac function in splenectomized sham or TAC mice was assessed 4 w after surgery, in order to define the long-term effects of splenectomy on LV remodeling in non-ischemic HF.

### Statistical analysis

All data are mean ± SD. Statistical comparisons between sham and TAC groups were performed using the unpaired Student’s t-test or two-way ANOVA, as appropriate. A p value of < 0.05 was considered significant.

## Results

### TAC induces cardiac remodeling and failure

**Fig 1:**
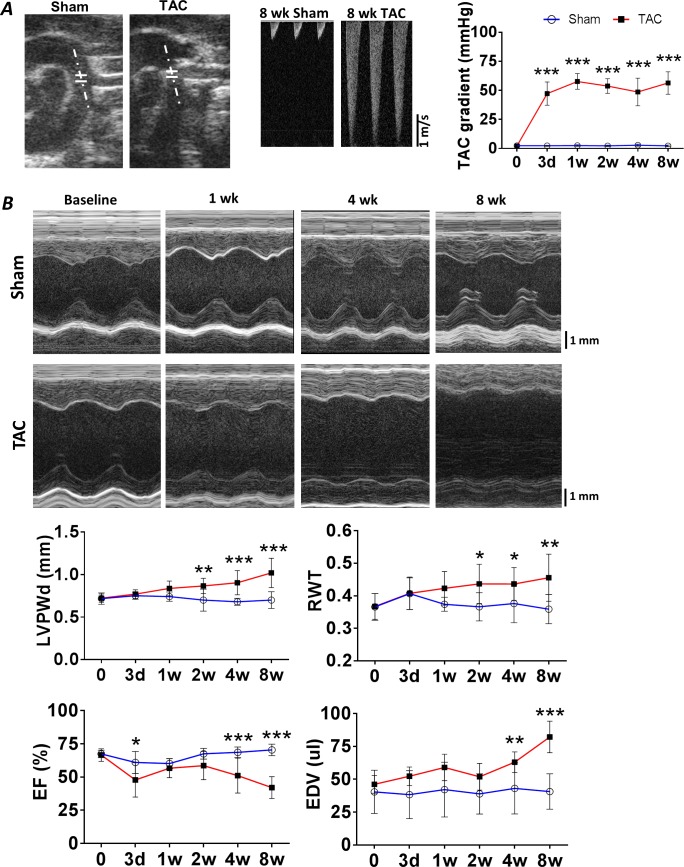
TAC-induced cardiac remodeling. ***A*,** *Left*, Representative 2D images and Doppler traces across the aortic arch in sham-operated and TAC mice at 8 w after surgery demonstrating marked elevated blood flow velocity in TAC mice. *Right*, corresponding group data for the pressure gradient in the same groups measured serially over time. ***B***, Representative M-mode echocardiograms performed at various time points after TAC or sham surgery, along with group data for LV posterior wall thickness at end-diastole (LVPWd), relative wall thickness (RWT, 2 * LVPWd/end-diastolic diameter), LV end-diastolic volume (EDV), and LV ejection Fraction (EF). n = 5–6 in sham groups and n = 6–8 in TAC groups. *p<0.05, **p<0.01, ***p<0.001 vs. sham.

Serial echocardiography was performed to evaluate LV structure and function from 72 h to 8 w after TAC or sham operation. Over 8 w, TAC resulted in a stable and significant increase in the pressure gradient measured across the aortic arch (~50–60 mm Hg), suggesting consistent pressure loading of the LV (Fig 1A). As illustrated by the representative M-mode images and summary data in Fig 1B, at 2 w after TAC, mice exhibited concentric LV hypertrophy (increased wall thickness) but maintained chamber size (EDV, end-diastolic volume) and ejection fraction (EF) as compared with sham-operated mice. Beyond this time frame, from 4 to 8 w, TAC mice exhibited progressive LV dilatation, systolic dysfunction and more eccentric hypertrophy without further increase in the relative wall thickness (RWT). Gravimetric data at 8 w after surgery (Table 1) indicated significant hypertrophy of the LV and the atria in TAC mice as compared with sham mice, together with increased lung and right ventricular (RV) weight, suggesting HF with pulmonary congestion and secondary RV hypertrophy. Normalized splenic weight was also significantly increased in TAC mice, whereas liver weight was unchanged.

**Table 1:** Gravimetric measurements in 8 w TAC and sham mice.

Parameter	Sham (n = 5)	TAC (n = 6)
**BW (g)**	26.9 ± 3.8	28.8 ± 2.1
**TL (mm)**	17.6 ± 0.5	17.1 ± 0.2
**Heart/TL (mg/mm)**	7.83 ± 0.81	10.63 ± 1.28*
**Atria/TL (mg/mm)**	0.51 ± 0.02	0.70 ± 0.06 *
**RV/TL (mg/mm)**	1.36 ± 0.04	1.57 ± 0.06 *
**LV/TL (mg/mm)**	5.74 ± 0.34	8.32 ± 0.45 *
**Spleen/TL (mg/mm)**	4.33 ± 0.09	4.95 ± 0.19 *
**Lung/TL (mg/mm)**	9.87 ± 0.67	13.98 ± 1.72 *
**Liver/TL (mg/mm)**	80.81 ± 5.24	82.34 ± 1.31

BW, body weight; TL, tibia length; RV, right ventricle; and LV, left ventricle.

*p < 0.05 vs. sham.

### Expansion of cardiac macrophages and dendritic cells after pressure-overload

**Fig 2:**
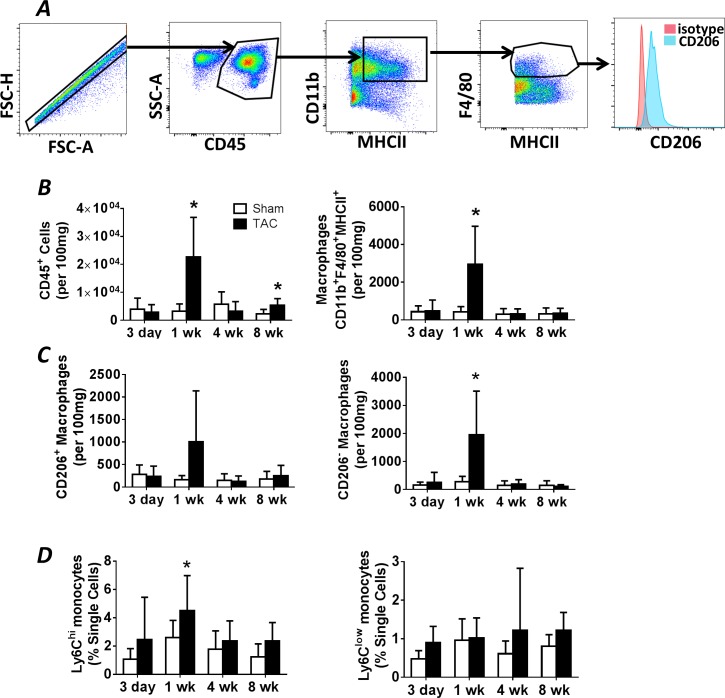
Cardiac macrophage abundance during pressure-overload remodeling. ***A*,** Representative gating strategy used to identify cardiac macrophages, as well as CD206^+^ and CD206^−^ macrophage subsets, in mouse hearts. ***B***, Quantitation of flow cytometry data collected from cardiac mononuclear cell isolates at the indicated time points after TAC or sham operation, including total CD45^+^ leukocytes, total CD45^+^CD11b^+^F480^+^MHCII^+^ macrophages, and ***C*,** CD206^−^ (M1) and CD206^+^ (M2) macrophages. ***D*,** Quantitation of flow cytometry data for Lin^–^CD11b^+^Ly6C^hi^ and Lin^–^CD11b^+^Ly6C^low^ peripheral blood monocytes at the indicated time points. n = 6–10 in sham groups; n = 6–12 in TAC groups. *p<0.05 vs. sham.

We evaluated MPs in the blood, spleen, and heart during the development of pressure-overload hypertrophy and failure. Fig 2A depicts the flow cytometry gating strategy used to identify CD45^+^CD11b^+^MHCII^+^F4/80^+^cardiac macrophages, with further sub-division into CD206^−^ pro-inflammatory ‘M1 polarized’ and CD206^+^ reparative ‘M2 polarized’ cells [14, 16, 19]. Fig 2B and 2C show group data for total CD45^+^ leukocytes, and total, M1, and M2 macrophages in the heart during pressure-overload. Total leukocytes and macrophages in the heart, assessed by absolute quantitation, increased early (1 w) after TAC (as compared with sham) during the compensated stage (prior to the transition to failure) with subsequent normalization. Of the CD45^+^CD11b^+^MHCII^+^F4/80^+^ macrophage population, CD206^+^ cells were not significantly increased at any time point, whereas pro-inflammatory CD206^−^ cells were augmented 1 w after TAC (Fig 2C). Notably, total CD45^+^ leukocytes exhibited a smaller phasic increase late (8 w) after TAC during chronic pressure-overload HF, but without analogous changes in macrophage populations.

We next evaluated whether changes in the cardiac macrophage profile during pressure-overload were accompanied by changes in blood monocytes. As seen in Fig 2D, circulating pro-inflammatory CD11b^+^Ly6C^hi^ monocytes, but not Ly6C^low^ monocytes, were significantly increased only at 1 w after TAC as compared with sham, with comparable levels between the groups thereafter. Splenic Lin^−^CD11b^+^Ly6C^hi^ and Lin^−^CD11b^+^Ly6C^low^ monocyte abundance is shown in S1 Fig. In contrast to circulating levels, splenic Ly6C-expressing monocytes were not impacted during the development of pressure-overload HF, with levels comparable to those observed in sham-operated mice both early and late after TAC.

**Fig 3:**
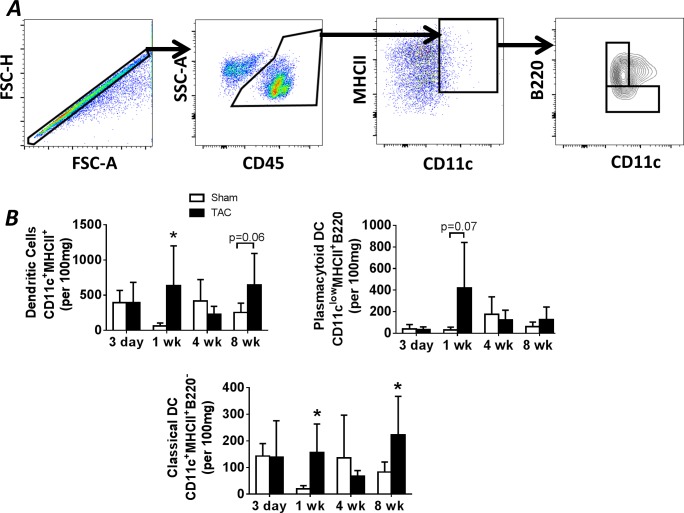
Dendritic cell populations in the heart during pressure-overload remodeling. ***A*,** Representative gating strategy used to identify cardiac DCs, including plasmacytoid and classical DCs, in the same mouse hearts as in Fig 2. ***B***, Quantitation of flow cytometry data from the heart at the indicated time points after TAC or sham operation, including total CD45^+^CD11c^+^MHCII^+^ DCs, CD11c^low^MHCII^+^B220^+^ plasmacytoid DCs, and CD11c^+^MHCII^+^B220^−^ classical DCs. *p<0.05 vs. sham.

The innate immune response also involves the activation of DCs that orchestrate antigen presentation to T-cells [10]. Fig 3A depicts the gating strategy used to identify CD45^+^CD11c^+^MHCII^+^ DCs, which were further separated into CD11c^+^MHCII^+^B220^−^ classical DCs and CD11c^low^MHCII^+^B220^+^ plasmacytoid DCs [16, 20, 21]. As seen in the group data in Fig 3B, in concert with increased cardiac macrophages early after pressure-overload, cardiac DC abundance was increased 1 w after TAC, as compared with sham mice. Unlike cardiac macrophages, a second DC increase, nearly statistically significant, was observed late (8 w) after TAC. Examination of the two major DC subsets revealed augmented levels of classical DCs at both 1 and 8 w after TAC, as compared with sham mice (a trend towards increased levels was observed for plasmacytoid DCs at 1 w). Circulating and splenic DCs were measured in a similar fashion using flow cytometry. As seen in S2 Fig, no differences were observed in classical or plasmacytoid DCs in the blood or spleen of TAC mice during the progression of pressure-overload hypertrophy and HF, suggesting local proliferation of DCs in the hearts of TAC mice.

### Global MP ablation after the development of pressure-overload hypertrophy does not delay the transition to HF

**Fig 4:**
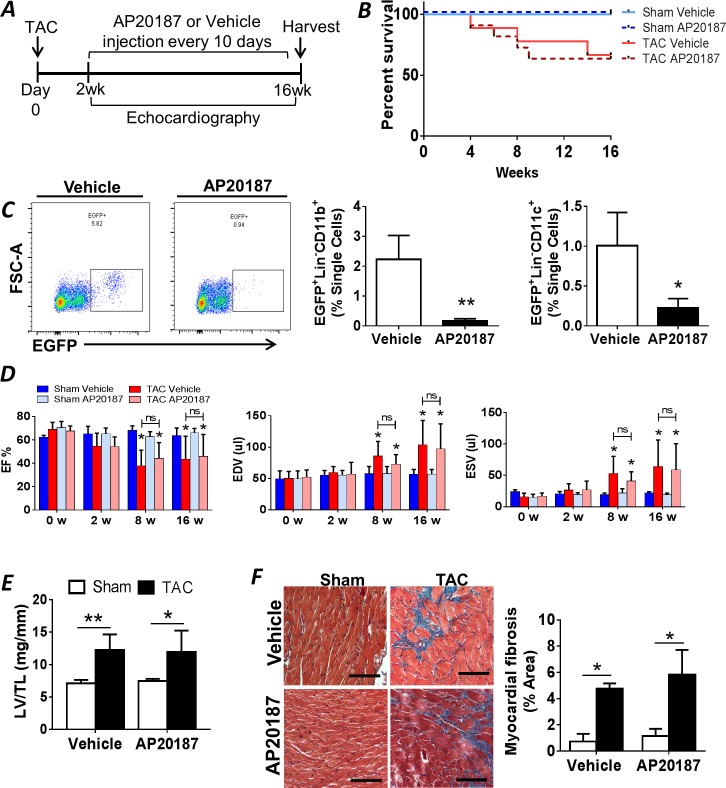
Mononuclear phagocyte depletion during pressure-overload in MaFIA mice. ***A*,** Schematic of the experimental protocol for periodic macrophage and DC ablation in MaFIA mice starting 2 w after TAC or sham operation. ***B***, Kaplan-Meier survival curves for MaFIA mice treated with either AP20187 or vehicle after TAC or sham operation (n = 5 for sham groups, n = 7 for TAC groups). ***C*,** Representative FACS plots of EGFP^+^ cells in peripheral blood from MaFIA mice 3 d after AP20187 or vehicle treatment, and corresponding quantitation of EGFP^+^Lin^−^CD11b^+^ monocytes and EGFP^+^Lin^−^CD11c^+^ dendritic cells (n = 5 per group), *p<0.05, **p<0.01 vs. vehicle controls. ***D*,** Echocardiographic parameters in sham-operated and TAC MaFIA mice treated with either vehicle or AP20187 as per the protocol in panel A. EF, ejection fraction; EDV, end-diastolic volume; ESV, end-systolic volume. ***E*,** LV gravimetric data for sham and TAC MaFIA mice treated with vehicle or AP20187; TL, tibia length. ***F*,** Representative Masson trichrome stains of LV sections 16 w after TAC or sham surgery in MaFIA mice treated with either vehicle or AP20187, and quantitation of tissue fibrosis. Scale bar, 100 μm. For panels ***D-F***, n = 5 in sham groups, n = 7–8 in TAC groups. *p < 0.05, **p<0.01 vs. respective sham group.

Having profiled cardiac macrophages and DCs after TAC, we next evaluated their functional impact on cardiac remodeling using MaFIA mice. MaFIA mice express a transgene comprised of EGFP and a suicide construct (human low-affinity nerve growth factor receptor-FK506-binding protein-Fas cytoplasmic domain) downstream of the *c-fms* promoter [24]. This promoter drives the expression of colony-stimulating factor 1 receptor (CSF1R), which is expressed by macrophages and DCs. Administration of the drug AP20187 dimerizes the cytoplasmic Fas fragments to induce Fas-induced apoptosis of MPs [24]. Beginning 2 w after TAC or sham surgery, mice were administered either AP20187 (0.5 mg/kg i.p.) for periodic MP depletion or vehicle control every 10 d and followed for 14 w (Fig 4A). We specifically depleted MPs after the development of compensatory hypertrophy to determine their functional role during the transition to HF and progression of systolic dysfunction. AP20187 dosing was based on preliminary experiments showing maximal macrophage and DC ablation in the blood at 3 d post-injection with normalization to baseline levels by 10 d.

Over the treatment period, mortality was similar between AP20187 and vehicle treated TAC groups (Fig 4B). AP20187 treated mice lost ~10% body weight at 3 d following treatment; however, body weights normalized and were comparable to vehicle-treated mice by 8 d following injection, and there were no significant differences in body weight between groups at the end of the study. MP depletion efficacy was confirmed by measuring circulating monocytes and DCs in the peripheral blood. AP20187 treatment depleted ~90% of Lin^−^CD11b^+^EGFP^+^ blood monocytes and ~75% of Lin^−^CD11c^+^EGFP^+^ DCs 72 h after injection (Fig 4C). However, serial depletion of monocyte/macrophages and DCs over 14 w did not alter the progression of LV dilatation, systolic dysfunction, and cardiac remodeling. Specifically, as compared with the TAC-vehicle group, the TAC-AP20187 group exhibited similar reductions in LVEF, comparable increases in LVEDV and LVESV *(*Fig 4D*)*, and no differences in the degree of LV hypertrophy (normalized LV weight) and cardiac fibrosis (trichrome staining) (Fig 4E and 4F). There was no impact of AP20187 on any of these parameters in the sham-operated mice.

### The cardiosplenic axis is not required for the progression of chronic pressure-overload HF

**Fig 5:**
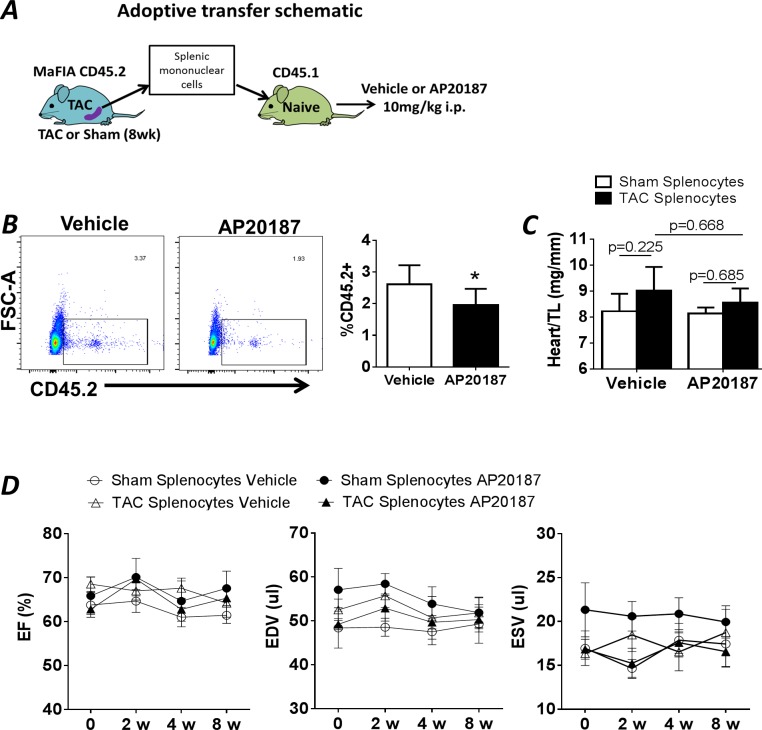
Adoptive transfer of splenocytes from sham-operated and TAC mice. ***A*,** Schematic of protocol for the adoptive transfer of splenocytes, harvested from CD45.2^+^ MaFIA mice 8 w after TAC or sham operation, into naïve CD45.1^+^ recipient mice. AP20187 or vehicle was administered immediately after transfer. ***B*,** Representative FACS plots of blood from recipient mice 24 h after adoptive transfer and administration of either vehicle or AP20187, and corresponding group data for CD45.2^+^ cells (n = 10/group). *p<0.05 vs. vehicle. ***C*,** Heart weight normalized to tibia length in recipient mice 8 w after adoptive transfer of either sham or TAC splenocytes, with or without concomitant AP20187 (n = 5/group). ***D*,** Echocardiographic parameters in recipient mice, over the 8 w course after adoptive transfer. EF, ejection fraction; EDV and ESV, end-diastolic and end-systolic volume.

The above data suggested that mononuclear phagocytes were dispensable for remodeling progression during chronic pressure-overload HF. Nonetheless, as we observed splenic hypertrophy (Table 1) and an early circulation of pro-inflammatory monocytes in TAC mice, and as we have previously shown the importance of a pathological pro-inflammatory cardiosplenic axis during cardiac remodeling chronic ischemic HF [16], we further evaluated the role of the spleen in TAC-induced non-ischemic HF. Splenic mononuclear cells were isolated from CD45.2 MaFIA mice 8 w after TAC or sham operation and adoptively transferred into WT CD45.1 C57BL/6 recipient mice that were then followed for another 8 w (Fig 5A). To isolate the role of splenic MPs, recipient mice received a single high dose (10mg/kg i.p.) of AP20187 immediately after transfer to ablate donor monocytes, macrophages, and DCs (vehicle control). Flow analysis for circulating CD45.2^+^ cells in recipient mice 24 h after adoptive transfer revealed ~2.6% of total monocytes/lymphocytes were donor CD45.2^+^ cells, which was significantly decreased by ~25% in recipient mice that received AP20187 (Fig 5B). Over the 8-week follow-up period, cardiac structure and function were preserved and comparable in all groups of recipient mice, with no significant differences in heart weight (Fig 5C), or LV size and systolic function (Fig 5D). Moreover, sham-operated and TAC donor MaFIA mice were followed for an additional 4 w after survival splenectomy (SPX), and compared to a parallel group of TAC mice with sham splenectomy (TAC nSPX group). There were no differences in LV structure or systolic function between the TAC SPX and nSPX groups, nor did SPX impact LVEF, EDV, or ESV in TAC mice (S3 Fig).

## Discussion

There are several key findings of our study. First, we have shown that there is a phasic expansion of circulating LyC6^hi^ monocytes, and pro-inflammatory CD206^−^ macrophages and classical DCs in the heart, during mechanical pressure-overload. Pro-inflammatory monocyte and macrophage expansion occurs during early remodeling, prior to the development of significant hypertrophy and systolic dysfunction, and resolves late during chronic HF. In contrast, classical DC expansion is biphasic occurring both early, prior to systolic dysfunction, as well as late, during established HF. Second, cardiac MP expansion occurs without significant expansion of DCs in the blood, or Ly6C^+^ monocytes and DCs in the spleen. Third, chronic depletion of MPs initiated at the compensated hypertrophy stage did not alter the subsequent course of LV remodeling and late HF. Fourth, the combination of adoptive transfer and splenectomy studies established that, unlike in chronic ischemic HF [16], a pathological cardiosplenic axis does not play a significant role in pressure-overload HF. Taken together, we conclude that mononuclear phagocytes are dispensable for the progression of pressure-overload HF, once significant cardiac hypertrophy and normalization of circulating pro-inflammatory monocytes are apparent.

While inflammatory activation is considered a central component of cardiac remodeling in response to pressure-overload [5–8], the role of specific immune cell populations has not been well characterized. Recent studies focusing on adaptive immune cells [4, 25] have demonstrated an essential role for T-cell activation and specific antigen recognition in the pathogenesis of cardiac remodeling and chronic HF during pressure-overload. By extension, T-cell activation implies a key role for macrophages and dendritic cells, as these cells mediate antigen presentation [10, 20, 26]. In this regard, while prior studies have indeed shown cardiac macrophage accumulation in non-pharmacological models of pressure-overload [11, 12], its precise role in hypertrophic remodeling and the transition to HF is less clear. Moreover, no studies to our knowledge have examined DCs in the pressure-overloaded heart.

After TAC, the time course of cardiac remodeling was characterized by a very early (at 3 d) decline in LVEF in the absence of adaptive hypertrophy, followed by LVEF recovery at 1 w and a compensated stage of concentric hypertrophy and preserved LVEF up to 2 w. Transition to LV systolic dysfunction and chamber dilatation and HF was observed by 4 w, with further deterioration of systolic function in addition to pulmonary edema at 8 w. Paralleling these functional stages, we observed Ly6C^hi^ monocytosis (but no increase in DCs) in the peripheral blood, and pro-inflammatory CD206^−^ macrophage and classical DC accumulation the heart, occurring early, by 1 w after TAC. Following this, macrophage levels normalized and were comparable to sham-operated hearts by 4–8 w, during the transition to HF. This suggests that circulating monocytes (presumably derived from the bone marrow as there were no changes observed in the spleen) contribute to cardiac macrophage accumulation early during pressure-overload, whereas cardiac DC populations may augment primarily via local proliferation. Additionally, we observe a second phase of expansion for both total leukocytes and classical DC in the heart late (8 w) after TAC. Given that DCs are potent activators of T-cells, this late increase is consistent with prior studies demonstrating a crucial role for activated T-cells during chronic pressure-overload HF [4, 25].

Prior work has established that cardiac macrophages in normal, naïve mice resemble an M2-like phenotype [19], which is typically associated with anti-inflammatory properties [27]. Our findings of greater accumulation of CD206^−^ macrophages during pressure-overload suggest a switch to a pro-inflammatory M1 macrophage phenotype that can propagate tissue damage through the production of proteases, cytokines and free radicals [28]. The increase in cardiac macrophages early after TAC is consistent with previous reports [11, 12], although categorization of macrophages based on F4/80 and Ly6C by Weisheit et al [12] suggested expansion of a predominantly Ly6C^low^ M2-type population, rather than a Ly6C^hi^ M1-type population. The reason for the discrepancy between our results and the study by Weisheit et al is not entirely clear, but may be related to differences in gating strategy and perhaps differences in gender (female mice were exclusively used). Nonetheless, Xia et al [11] have shown that the cardiac cytokine and chemokine gene expression profile early in TAC favors a pro-inflammatory milieu (i.e., upregulation of TNF, IL-1β, and monocyte chemoattractant protein-1), which is more consistent with CD206^−^ macrophage predominance. Further, DCs primarily function as antigen presenting cells, providing the stimulus for T-cell activation, suggesting that cardiac DC accumulation is a proximate event required for the activation of T-cells shown to mediate, at least in part, the transition to failure during chronic pressure-overload [4, 25].

To specifically determine the importance of MPs during pressure-overload remodeling and HF, we used transgenic MaFIA mice in which all CSF1R expressing cells (*i*.*e*., all MPs) can be reversibly depleted in an inducible and graded fashion [24]. We induced MP depletion beginning 2 w after TAC, a time point just prior to the transition to HF when there was hypertrophy but no overt systolic dysfunction. Interestingly, despite repeated depletion of macrophages and DCs up to 16 w post-TAC, remodeling progression and the transition to failure was unabated. These results indicate that once there is significant cardiac hypertrophy evident in response to pressure-overload, and normalization of circulating pro-inflammatory monocytes, mononuclear phagocytes are not critical for the progression to HF. This was a paradoxical result, given that depletion of MPs in ischemic and lipotoxic models of cardiac injury significantly modulates the progression of cardiac dysfunction [14, 29, 30]. However, our data do not exclude the possibility that therapeutic manipulation of macrophages and/or DCs at earlier stages of pressure-overload influence subsequent remodeling progression. Moreover, the suicide construct in MaFIA mice indiscriminately targets all MP populations. It is possible that more specific targeting of discrete MP subsets (e.g., CD206^−^ macrophages or Ly6C^hi^ monocytes), or individual signaling pathways in MPs, could yield considerably different results. For example, prior studies have shown that mineralocorticoid receptor (MR) signaling in myeloid cells is essential for adverse LV remodeling in response to mechanical or pharmacological pressure-overload [31, 32]. The cardioprotective effects of MR deficiency in myeloid cells was attributed to augmentation of alternative M2 macrophage activation [31] and concomitant reduction of cardiac inflammation in pressure-overloaded MR knockout mice [31, 32].

In chronic ischemic HF, we previously reported profound splenic remodeling, splenocyte activation, and homing of tissue-injurious splenic immune cells to the heart, all indicating a pro-inflammatory cardiosplenic axis [16]. Deployment of cells from the spleen to sites of inflammation has also been documented in models of stroke and acute myocardial infarction [33, 34]. In our study of chronic pressure-overload HF, despite splenic hypertrophy, there was no expansion of splenic Ly6C^+^ monocyte and DC populations. Moreover, our HF splenocyte adoptive transfer and splenectomy studies did not support an important role for immune cell trafficking from the spleen to the heart in established pressure-overload HF. These findings are in contrast to chronic ischemic HF [16], during which robustly activated splenocytes traffic to the heart to promote fibrosis and adverse remodeling. Hence, the biological activity and tissue profile of inflammatory cells differs significantly between ischemic and non-ischemic etiologies of HF. This may be related to significant differences in the degree of tissue injury and cardiac cell death between the two models, which would be expected to produce vastly different stimuli to the immune system during HF development.

Innate immune cells such as macrophages may accumulate in inflamed tissue via external recruitment from the bone marrow and spleen, or via local proliferation of tissue resident cells. While the spleen serves as a reservoir for immune cells that can be rapidly mobilized after injury, recent fate-mapping studies in mice have shown that under steady-state conditions, resident cardiac macrophages are derived from differing ontological lineages with varying functions [35]. Disruption of the steady state by cardiac stress alters macrophage composition with both expansion of resident cells and infiltration of monocyte-derived cells [36, 37]. These subsets are postulated to have different functional roles, with resident cells promoting tissue repair and infiltrating cells exacerbating inflammation and tissue injury. One limitation of our study is that we did not characterize this dichotomy of resident versus infiltrating macrophage phenotype in our studies. Hence, although our results indicated no overall expansion of cardiac mononuclear phagocytes in the chronic stage of pressure-overload HF, it is possible that pressure-overload remodeling is accompanied by an altered distribution of resident versus infiltrating cells.

In summary, we have shown that during mechanical pressure-overload, there is a circumscribed expansion of circulating Ly6C^hi^ monocytes, and cardiac CD206^−^ macrophages that mainly occurs early during remodeling before the development of significant hypertrophy and systolic dysfunction, and which resolves during chronic HF. In contrast, classical DCs accumulate in the pressure-overloaded heart in a biphasic manner both early (analogous to macrophages), but also late during chronic established HF. This accumulation of cardiac mononuclear phagocytes occurs without significant alterations of splenic monocytes and DCs and in the absence of a pro-inflammatory cardiosplenic axis. Moreover, mononuclear phagocytes are not critical for the progression of pressure-overload HF once significant cardiac hypertrophy and normalization of circulating monocytes have occurred. This profile of immune cell activation is markedly different than chronic ischemic HF, and suggest that different etiologies of HF require different approaches to therapeutic immunomodulation to improve pathological cardiac remodeling.

## Supporting Information

S1 Fig Splenic monocyte populations during cardiac pressure-overload. ***A*.** Representative flow cytometry gating strategy for splenic Ly6C^+^ monocytes ***B*.** Quantitative group data for overall monocytes (Lin^−^CD11b^+^), pro-inflammatory monocytes (Lin^−^CD11b^+^Ly6C^hi^), and patrolling monocytes (Lin^−^CD11b^+^Ly6C^low^) in the spleen during the indicated time points after TAC or sham operation. Lin, lineage markers (CD90.2, CD49b, CD45R, NK1.1). n = 5–6 in Sham groups; n = 6–7 animals in TAC groups.
(TIF)

S2 Fig Circulating and splenic dendritic cell (DC) populations during cardiac pressure overload. ***A*,** Representative flow cytometry gating strategy for circulating and splenic DCs. ***B*,** Quantitative group data for DC subsets: Lin^−^CD11c^+^ total DCs, Lin^−^CD11c^low^B220^+^ plasmacytoid DCs and Lin^−^CD11c^+^B220^−^ classical DCs during the indicated time points after TAC or sham operation. Top panels represent data from peripheral blood, and bottom panels represent data from spleen. Lin, lineage markers (CD90.2, CD49b, Ly6G, NK1.1). n = 5–6 in Sham groups; n = 6–7 animals in TAC groups.
(TIF)

S3 Fig Effects of splenectomy on cardiac remodeling in pressure-overload heart failure (HF). ***A*,** Schematic of experimental protocol. ***B*,** Kaplan-Meier survival curves for sham-operated and TAC mice with and without splenectomy (n = 5–6 per group). ***C-D*,** Quantitative group data for LV ejection fraction (EF), LV end-diastolic volume (EDV), end-systolic volume (ESV), and LV normalized to tibia length (TL) in sham-operated and TAC mice 4 w after splenectomy or sham splenectomy. SPX, splenectomy; nSPX, sham splenectomy.
(TIF)
