# Supplementary figures and images for: Mononuclear Phagocytes Are Dispensable for Cardiac Remodeling in Established Pressure-Overload Heart Failure
Source: PLoS One. 2017 Jan 26;12(1):e0170781. doi: 10.1371/journal.pone.0170781 (PMC5268479; doi:10.1371/journal.pone.0170781)

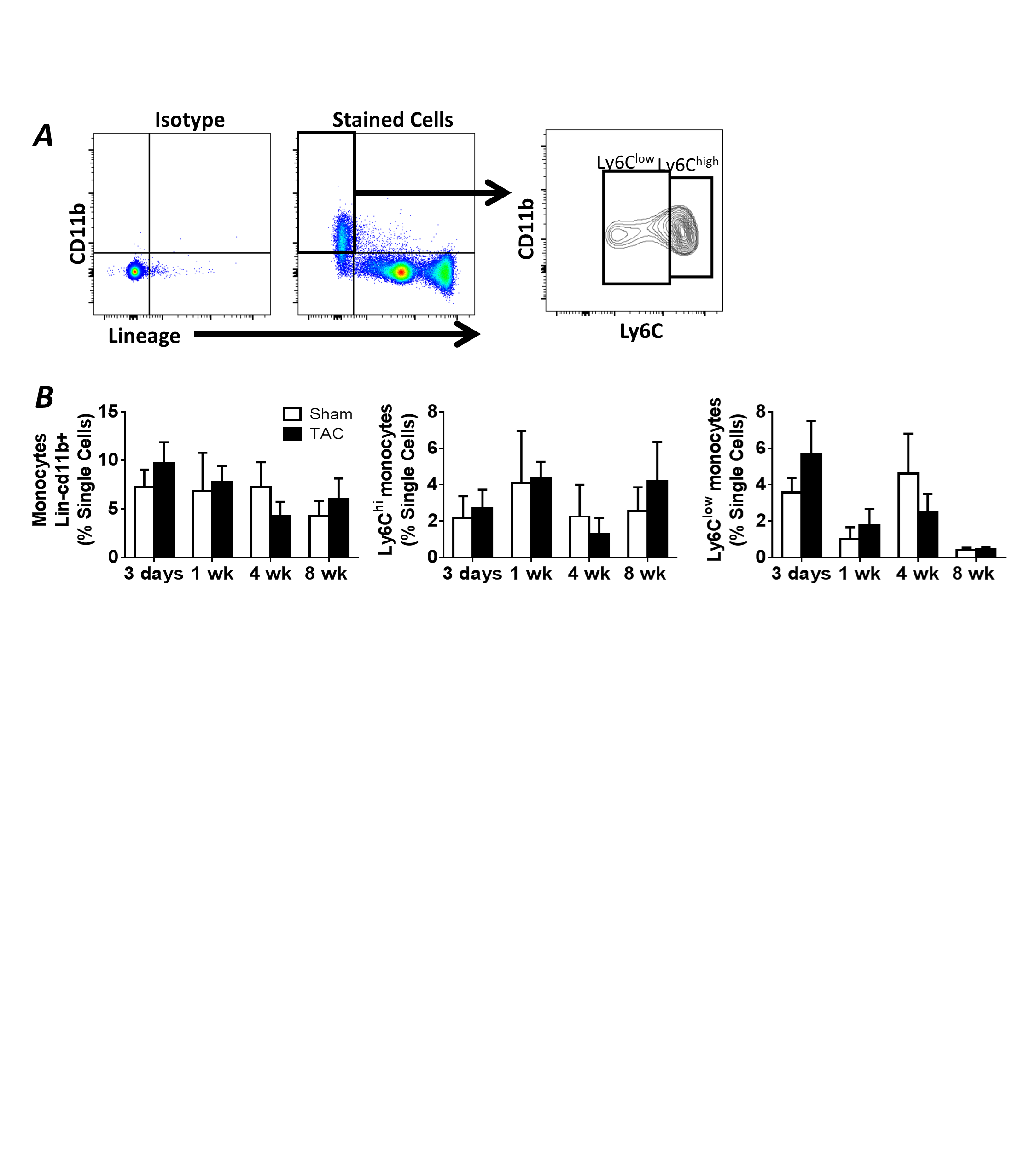

Supplement: S1 Fig — A. Representative flow cytometry gating strategy for splenic Ly6C+ monocytes B. Quantitative group data for overall monocytes (Lin−CD11b+), pro-inflammatory monocytes (Lin−CD11b+Ly6Chi), and patrolling monocytes (Lin−CD11b+Ly6Clow) in the spleen during the indicated time points after TAC or sham operation. Lin, lineage markers (CD90.2, CD49b, CD45R, NK1.1). n = 5–6 in Sham groups; n = 6–7 animals in TAC groups. (TIF) [file pone.0170781.s001.TIF]

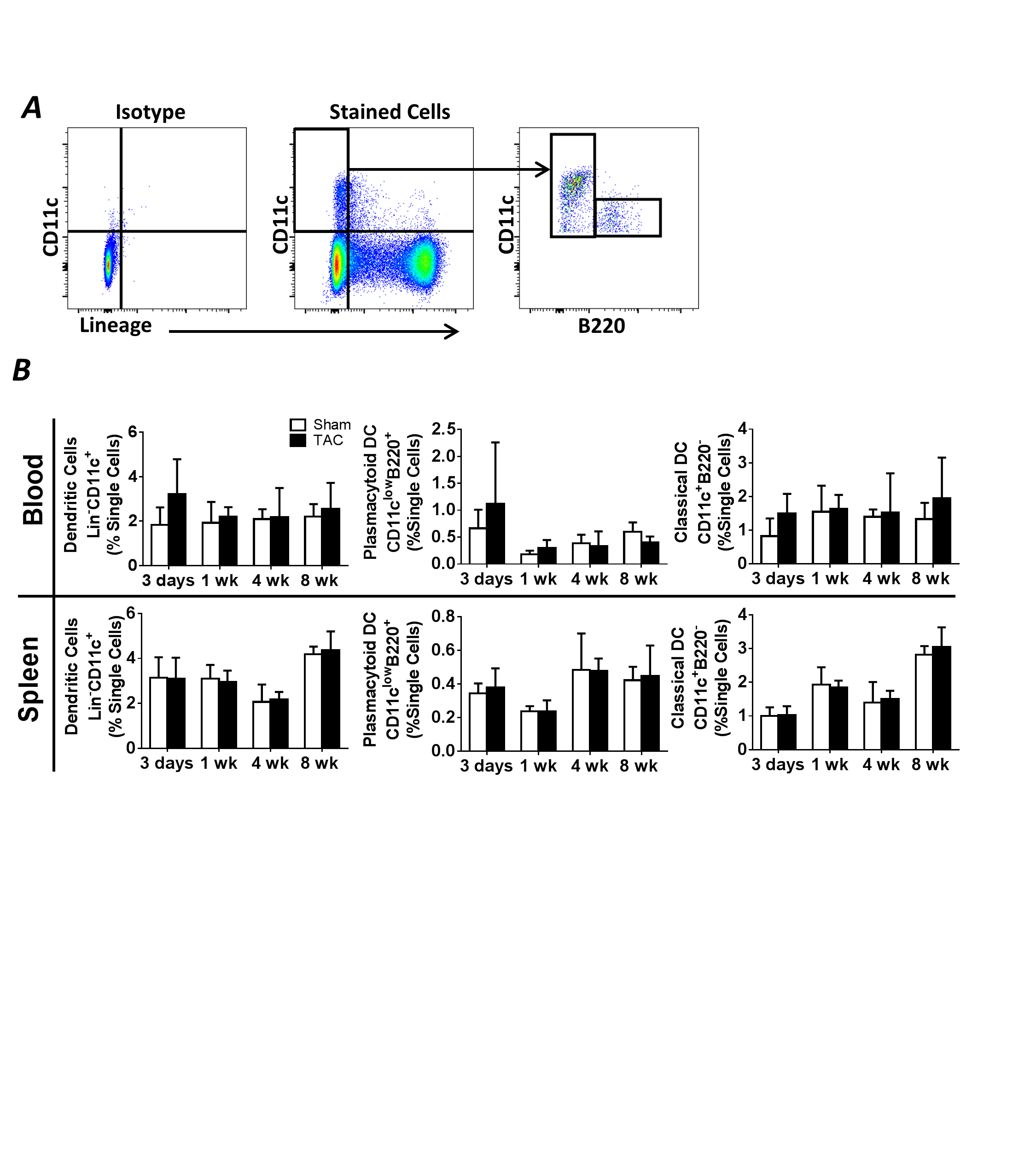

Supplement: S2 Fig — A, Representative flow cytometry gating strategy for circulating and splenic DCs. B, Quantitative group data for DC subsets: Lin−CD11c+ total DCs, Lin−CD11clowB220+ plasmacytoid DCs and Lin−CD11c+B220− classical DCs during the indicated time points after TAC or sham operation. Top panels represent data from peripheral blood, and bottom panels represent data from spleen. Lin, lineage markers (CD90.2, CD49b, Ly6G, NK1.1). n = 5–6 in Sham groups; n = 6–7 animals in TAC groups. (TIF) [file pone.0170781.s002.TIF]

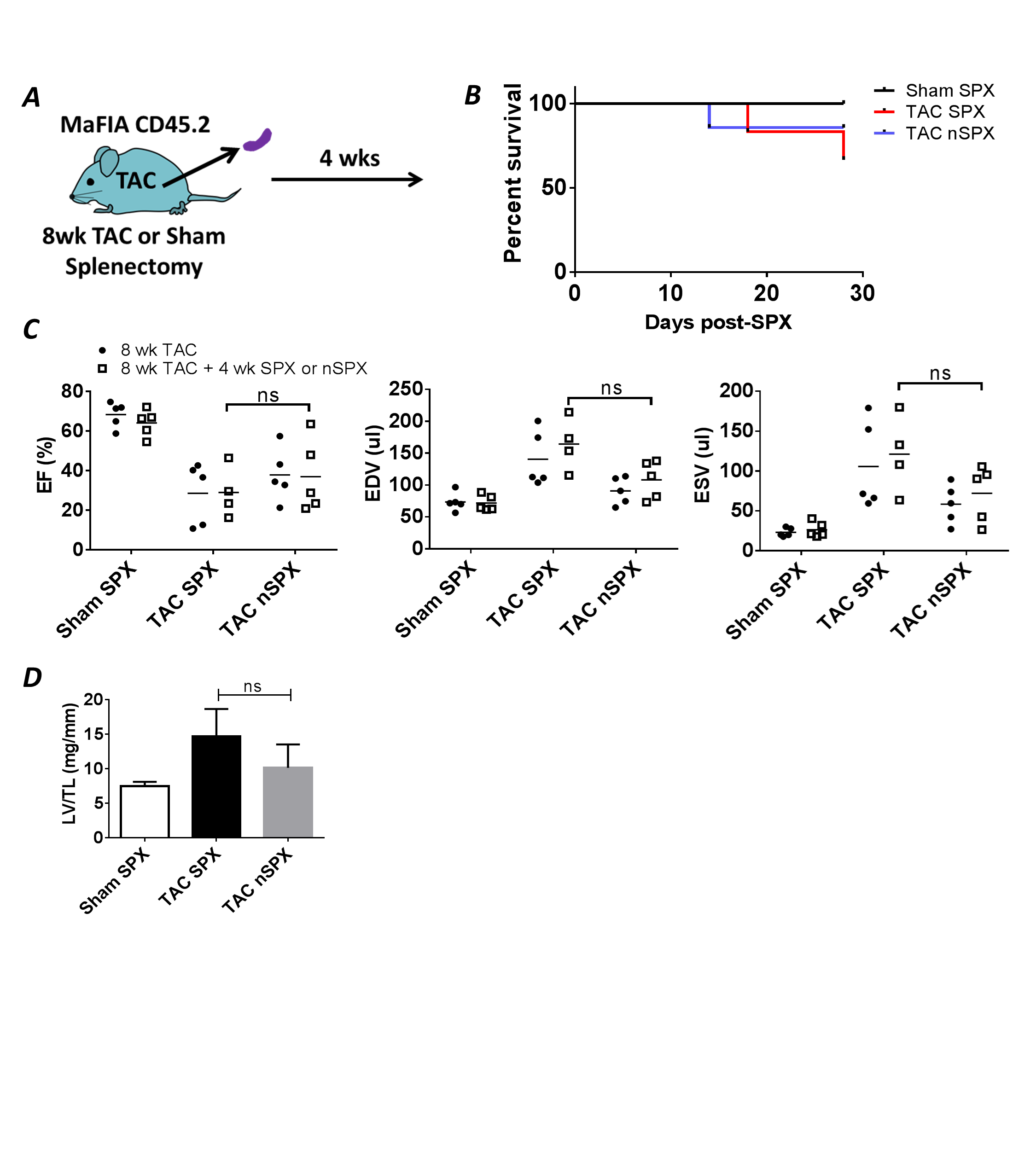

Supplement: S3 Fig — A, Schematic of experimental protocol. B, Kaplan-Meier survival curves for sham-operated and TAC mice with and without splenectomy (n = 5–6 per group). C-D, Quantitative group data for LV ejection fraction (EF), LV end-diastolic volume (EDV), end-systolic volume (ESV), and LV normalized to tibia length (TL) in sham-operated and TAC mice 4 w after splenectomy or sham splenectomy. SPX, splenectomy; nSPX, sham splenectomy. (TIF) [file pone.0170781.s003.TIF]
